# Early ambulation using a cycle ergometer on quadriceps muscle morphology in mechanically ventilated critically ILL patients in the intensive care unit: a randomized controlled trial

**DOI:** 10.1186/2197-425X-3-S1-A551

**Published:** 2015-10-01

**Authors:** LJ Santos, FA Lemos, T Bianchi, A Sachetti, AM Dall' Acqua, WS Naue, AS Dias, SR Vieira, MoVe-ICU Study Group

**Affiliations:** Universidade Federal do Rio Grande do Sul, Porto Alegre, Brazil

## Introduction

Patients in the intensive care unit (ICU) are exposed to prolonged immobility, which leads to loss of muscle mass. One resource that has proved to be of great utility in hospitals is the cycle ergometer, which is a stationary piece of equipment designed to enable cyclical rotations of lower and/or upper extremities and can be used to perform passive, active and resisted exercises.

## Objective

To evaluate and compare the effects of early ambulation using a bedside cycle ergometer with conventional physical therapy on the thickness and architecture of the quadriceps muscle in critically ill patients receiving invasive mechanical ventilation (IMV).

## Methods

Single-blind randomized controlled trial was conducted at Hospital de Clínicas de Porto Alegre (Brazil) ICU. Forty-two patients receiving IMV for 24 to 48 hours who were hospitalized for no longer than 1 week and had no restriction of lower limb movements. Interventions: After randomization, passive cycling exercise for the lower extremities was performed once daily for 20 minutes, at 20 revolutions per minute, until extubation or day 7 of the protocol plus conventional physical therapy in the intervention group. Bronchial hygiene maneuvers and passive exercises for the upper and lower extremities were performed twice daily for 30 minutes in both groups.

## Results

Thirty-two patients were included in the final analysis: 18 in the intervention group (52.3 ± 22.7 years) and 14 in the conventional group (56.1 ± 23.0 years). The interaction group*time showed no difference in the cross-sectional thickness of the quadriceps muscle (p = 0.100) or in the vastus lateralis fascicle length (p = 0.712), pennation angle (p = 0.603) and muscle thickness (p = 0.552) as assessed by ultrasound before and after the protocol.

## Conclusion

There was preservation of muscle thickness and architecture in the acute phase of ICU stay. However, the addition of exercise using a cycle ergometer to conventional physical therapy did not change the outcomes analyzed.

## References

[1] Burtin C, Clerckx B, Robbeets C, Ferdinande P, Langer D, Troosters T, Hermans G, Decramer M, Gosselink R (2009). Early exercise in critically ill patients enhances short-term functional recovery. Crit Care Med.

[2] Camargo Pires-Neto R, Fogaça Kawaguchi YM, Sayuri Hirota A, Fu C, Tanaka C, Caruso P, Park M, Ribeiro Carvalho CR (2013). Very early passive cycling exercise in mechanically ventilated critically ill patients: physiological and safety aspects--a case series. PLoS One.

